# Geographic Imputation of Missing Activity Space Data from Ecological Momentary Assessment (EMA) GPS Positions

**DOI:** 10.3390/ijerph15122740

**Published:** 2018-12-04

**Authors:** Jeremy Mennis, Michael Mason, Donna L. Coffman, Kevin Henry

**Affiliations:** 1 Department of Geography and Urban Studies, Temple University, Philadelphia, PA 19122, USA; kevinahenry@temple.edu; 2 Center for Behavioral Health Research, University of Tennessee, Knoxville, TN 37996, USA; mmason29@utk.edu; 3 Department of Epidemiology and Biostatistics, Temple University, Philadelphia, PA 19122, USA; donna.coffman@temple.edu

**Keywords:** missing data, spatial data, imputation, geographic imputation, activity space, ecological momentary assessment, EMA

## Abstract

This research presents a pilot study to develop and compare methods of geographic imputation for estimating the location of missing activity space data collected using geographic ecological momentary assessment (GEMA). As a demonstration, we use data from a previously published analysis of the effect of neighborhood disadvantage, captured at the U.S. Census Bureau tract level, on momentary psychological stress among a sample of 137 urban adolescents. We investigate the impact of listwise deletion on model results and test two geographic imputation techniques adapted for activity space data from hot deck and centroid imputation approaches. Our results indicate that listwise deletion can bias estimates of place effects on health, and that these impacts are mitigated by the use of geographic imputation, particularly regarding inflation of the standard errors. These geographic imputation techniques may be extended in future research by incorporating approaches from the non-spatial imputation literature as well as from conventional geographic imputation and spatial interpolation research that focus on non-activity space data.

## 1. Introduction

The role of space and place in shaping health has received increasing attention in the health and medical research community [1,2,3]. Researchers have argued, however, that investigating the influence of place on health should focus not simply on an individual’s residential neighborhood, but on the environmental exposures that occur throughout an individual’s activity space [4,5,6]—the routine locations an individual visits throughout his or her daily life, such as places of work or school, recreation and leisure, social interaction with friends and family, and so on [7]. Activity space is recognized as a key construct in investigations of substance use, physical activity, stress, healthy eating, exposure to air pollution, and other place-related health outcomes [8,9,10,11,12]. Research on activity space and health has been facilitated by the development and adoption of geospatial technologies, such as global positioning systems (GPS), which allows for the tracking and encoding of individual mobility, and geographic information systems (GIS), which facilitates the integration of mobility data with other spatial data capturing exposures to environmental health hazards or amenities [13,14].

One novel approach to capturing activity space data for studies of place and health behaviors combines GPS with ecological momentary assessment (EMA), a technique where individuals complete brief surveys concerning their moods, behaviors, and social interactions, typically via a mobile phone. EMA has the advantage of capturing ‘momentary’ health data in real-time and in individuals’ natural environments, thus helping to reduce bias associated with survey recall [15]. GPS can be used to simultaneously capture location at the moment of EMA survey response, thus yielding a set of georeferenced EMA observations, each with a precise location and time-stamp. In sum, these EMA location data can be considered an expression of an individual’s activity space and can be combined with other spatial data within GIS [16], an approach termed geographic EMA (GEMA).

GEMA has been used to investigate a variety of health behaviors, but perhaps most prominently for studies of place effects on substance use, where activity space data are typically used to assess individuals’ exposures to environmental risk, such as neighborhood disadvantage, neighborhood disorder, or the locations of stores selling alcohol or tobacco, to develop statistical models of the effect of such exposures on substance use and related outcomes [17,18,19,20]. One particular challenge with the use of GEMA concerns the occurrence of missing activity space data, i.e., situations in which an EMA survey response is recorded, but a corresponding geographic coordinate position is not captured. GPS-derived coordinate positions may be unobtainable due to a variety of reasons: Atmospheric refraction of the signal from the satellite to the receiver; the presence of tree canopy, buildings, or other features, which can serve to block or attenuate communication between the satellite and receiver; the GPS signal may reflect off buildings or other features in the environment, generating multiple paths from the satellite to the receiver; error in the synchronization of the satellite and receiver clocks; error in the ephemeris information concerning the satellite orbital position; and the geometric dilution of precision (GDOP) regarding the geometric relationships that occur between the receiver and satellites, where a coordinate position is more difficult to obtain when satellites cluster together in the sky, and which is typically encoded during GPS data capture using National Marine Electronics Association (NMEA) data standards [21,22]. Consequently, missing or inaccurate activity space data in GEMA studies is a common occurrence [21,23]. 

The simplest approach to treating missing activity space data in GEMA is listwise deletion (i.e., complete case analysis)—the simple elimination of any EMA observation (i.e., an individual EMA response) with missing location data from the analysis. Unfortunately, this approach has the effect of reducing statistical power as well as introducing potential bias in geographic cluster detection and into measures of environmental exposure, and can result in biased regression parameter estimates and standard errors [24,25,26,27]. 

An alternative approach is to impute the missing activity space locations. Conventional statistical imputation for non-spatial data includes approaches that estimate missing data values using the mean of the known values or by replacing the missing value with a randomly selected known value [28,29]. Model-based approaches have employed regression or the expectation-maximization (EM) algorithm to estimate missing values [30,31]. More recently, multiple imputation methods have become more accessible and prominent, whereby model-based approaches are used to generate a distribution of missing data values, which can be employed in a series of analytical models and where parameter estimates are pooled [32,33].

Geographic imputation [34] departs somewhat from the statistical imputation literature and refers generally to estimating missing location data. It is typically applied to estimating residential locations for individuals from administrative health data in the case when address geocoding fails due to a missing or incorrect street address, or for other reasons [35,36,37]. Often this involves a situation where the residential municipality or zip code for an individual is known, and the analyst is attempting to estimate the residential location more precisely within that spatial unit, for instance, by estimating the location as the centroid (geometric center) of the unit or through an aerial interpolation technique that incorporates ancillary data to enhance the accuracy of the estimation [34,38,39,40,41,42].

Geographic imputation of missing activity space data, in the context of GEMA or similar data collection designs, differs from this previous geographic imputation research in several important ways: (1) For each individual, there are a set of activity space points (not just a single missing residential location); (2) the activity space data are not missing due to a failure of geocoding (i.e., there are no address data available); and (3) there is no spatial unit, such as a zip code, that constrains where the activity space point is to be estimated. Some researchers have also sought to impute missing GPS tracking data, i.e., a continuous stream of coordinate positions captured at regular time intervals, say, every 30 s, by taking a moving average of the known GPS locations [43], but this also differs from many conventional EMA study designs, which aim to sample at random, discrete moments in time, not continuously.

The aim of the present research is to develop and compare methods of geographic imputation for estimating the location of missing activity space data collected using GEMA. We consider this a pilot study that investigates the feasibility of adapting relatively simple geographic and conventional statistical (i.e., non-geographic) imputation methods to missing GEMA activity space data, and thus paves the way for the development of more sophisticated GEMA geographic imputation research in the future. The significance of this research lies in its ability to establish a baseline approach and comparative framework for geographic imputation of activity space data, which, to our knowledge, has not been addressed in the geographic imputation or statistical imputation literatures. The present research can also shed light on related domains of activity space data analysis other than GEMA methods where missing data may be problematic.

To this end, we use data from a previously published GEMA analysis of the effect of neighborhood disadvantage, captured at the U.S. Census Bureau tract level, on momentary psychological stress among a sample of urban adolescents [17]. Here, we aim to use geographic imputation to estimate the neighborhood disadvantage for EMA observations where momentary stress is recorded, but there is no associated geographic coordinate, and thus the neighborhood disadvantage at that EMA location is unknown. By holding out portions of the original data set, we investigate the impact of listwise deletion, as well as of two different geographic imputation methods we develop, on the direct and moderated effect estimates of neighborhood disadvantage on stress. 

## 2. Materials and Methods

### 2.1. Study Setting

Data are derived from the Social-Spatial Adolescent Study, a two-year longitudinal study of peer and environmental effects on substance use among an urban, primarily African American population of adolescents. The study follows 248 adolescents who were recruited primarily from a public adolescent medicine clinic in Richmond, Virginia between 2012 and 2014. Written informed assent was obtained from adolescents and consent was obtained from their parents prior to commencing any research activities. The Temple University, Virginia Commonwealth University, and the Richmond City Health Department’s institutional review boards approved the research protocol. All participants were given a mobile phone with embedded GPS for the duration of the study. Full battery assessments collecting home address, demographic information, substance use involvement, and other measures were completed in-person at baseline and every six months thereafter. In addition, every two months following enrollment, subjects received 3–6 EMA surveys per day over a four-day period via an embedded URL link to the survey sent as a text message to their mobile phone. The EMA survey asked questions about momentary moods, behaviors, and social interactions. During the moment of the EMA survey response, their location in the form of a geographic coordinate position was collected with GPS. The data set thus comprises a set of discrete activity space locations interspersed over one year for each subject (i.e., as opposed to, say, a GPS ‘track’ of individual movement taken over a smaller time frame). Further details on the study and the data can be found in [44,45].

### 2.2. Measures

**Figure 1:**
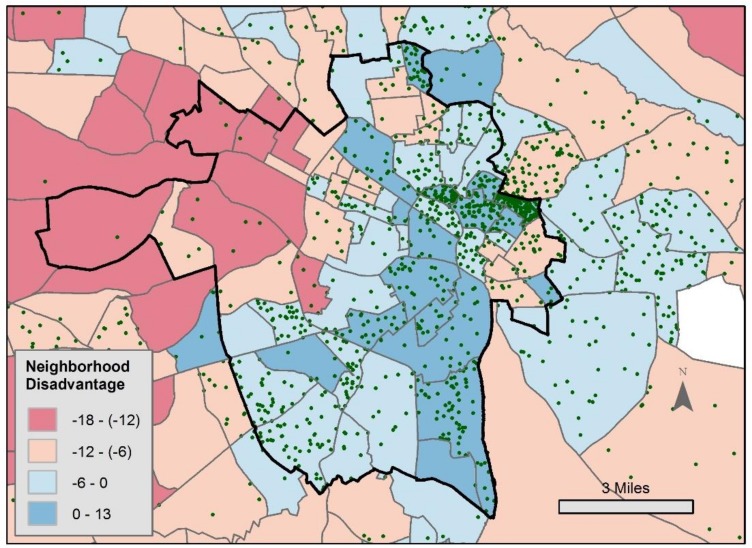
Map of the ecological momentary assessment (EMA) locations (green points) overlain on neighborhood disadvantage by census tract in the Richmond, Virginia area, where greater disadvantage is shown in blue. The city of Richmond is outlined in bold. The coordinate position of each EMA location is randomized within each tract for privacy protection. Note that some EMA locations occur outside the area of the map.

The present research investigates geographic imputation using the data and analyses from previously published research focusing on the association between exposure to neighborhood disadvantage and psychological stress [17]. These data were gathered in the first year of the study data collection. Of the 3882 EMA responses completed outside the home (as indicated by the subject in the EMA response), we used the 1617 for which coordinate location data were available and which occurred in the Richmond, Virginia study region, for 137 subjects (twelve EMA observations for two subjects were missing values of the stress outcome variable in the 1629 EMA observations reported in [17]). Figure 1 shows a map of the EMA locations located in and around the city of Richmond, with location randomized within each tract to preserve privacy. The use of this data set allows us to compare various imputation techniques in the context of a published, theoretically derived statistical model of a place association with a health outcome.

We focus on two particular statistical models presented in [17]. The first employs generalized estimating equations (GEE) to estimate the effect of neighborhood disadvantage on momentary stress (from the EMA) while controlling for age at enrollment (13 or 14 years), sex (male or female), race (white, African American, or other race), and substance use. Substance use was measured using the Adolescent Alcohol and Drug Involvement Scale (AADIS) [46], a continuous scale where higher values indicate greater substance use involvement. The AADIS has favorable internal consistency (Cronbach’s alpha = 0.94) and correlates highly with self-report and clinical measures of substance use (*r* = 0.72 and *r* = 0.75, respectively), and with subjects’ perceptions of the severity of their own substance use problem (*r* = 0.79). Age, sex, race, and substance use are taken from the full battery assessment collected at baseline. Stress is measured according to the EMA survey item “How stressed out are you right now?” with possible integer values of 1 (“Not at all stressed out”) through to 9 (“Very stressed out”).

Neighborhood disadvantage is conceptualized as a multidimensional measure incorporating aspects of income, educational attainment, residential stability, and family composition, as has been used in previous research [17,47]. For each EMA observation, neighborhood disadvantage is calculated based on the U.S. Census Bureau tract within which that EMA observation occurs. Tract disadvantage is calculated as ((*a*/10) + (*b*/10)) − ((*c*/10) + (*d*/10)), where *a* is the poverty rate, *b* is the percentage of female-headed households with children, *c* is the percentage of adults with a bachelor’s degree or higher, and *d* is the percentage of owner-occupied housing [47]. Higher values indicate greater neighborhood disadvantage (Figure 1). We calculate a measure, which we call ‘relative disadvantage’, which indicates whether the subject has traveled to an EMA location relatively more, or less, disadvantaged as compared to the tract where they reside. Relative disadvantage is calculated by subtracting the disadvantage measured at the subject’s home location (indicated at baseline) from the momentary disadvantage (disadvantage at the EMA location). The GEE model adjusts for subject and tract level clustering using an exchangeable correlation structure. Table 1 (Model 1) shows the results of the model reported in [17], where higher relative disadvantage is significantly associated with higher stress.

We also investigate a second GEE model, which includes a test of moderation. Here, we consider whether substance use involvement moderates the association of relative disadvantage with stress, where we expect the relationship to be stronger among subjects with greater substance use. For this purpose, we add an interaction term to Model 1 composed of the product of the substance use and relative disadvantage variables. The results are reported in Table 1 (Model 2), where the effect of relative disadvantage on stress is significantly greater for subjects with higher substance use involvement [17].

### 2.3. Analytic Plan

To support our analysis, we create three new data sets in which we retain a certain percentage of the original data set’s neighborhood disadvantage values and remove the rest, i.e., we ‘pretend’ the disadvantage values for the non-retained EMA observations are missing. For this purpose, we assigned each EMA observation a random number between 0 and 1, and then sorted them in ascending order. For the first new data set, we retained the first 50% (*n* = 808) of the disadvantage values, and for the second and third data sets, we retained the first 70% (*n* = 1131) and the first 90% (*n* = 1455), respectively. Thus, 50% of the disadvantage values are missing for the first data set, 30% are missing for the second data set, and 10% are missing for the third data set. The values that are set to missing are missing completely at random (MCAR) [48].

We use these data sets to test two geographic imputation methods. Note that our aim is not necessarily to impute a specific point location for each EMA observation with a missing location value, but rather to estimate a specific census tract location, which can serve as the basis for calculating a relative disadvantage value that can then be entered into the model of stress. The first imputation method is an adaptation of the hot deck (HD) imputation technique developed for non-geographic data [49] and is somewhat similar conceptually to the random property allocation technique described in [38]. In the conventional HD approach, a random value from the known values in the data set is used to replace a missing data value. We adapt this for GEMA activity space data by replacing a missing tract value for an EMA observation with a random tract, and thus a neighborhood disadvantage value, extracted from the retained (i.e., non-missing) values in the data set. Unlike with conventional HD imputation, however, the tract drawn from the retained values is extracted from an EMA observation for the same subject, rather than a random observation from the entire data set, i.e., the activity space location is imputed using another, previously visited activity space location. The neighborhood disadvantage value for the imputed tract is then used as an estimate for all the missing neighborhood disadvantage values for that subject.

We refer to the second geographic imputation method as activity space centroid (ASC) imputation. For each subject, the centroid (point location) of the known activity space locations for that subject is calculated as the mean of the X and Y geographic coordinates from the retained, non-missing, values in the data set, such that the centroid falls in the geometric center of the subject’s known EMA locations. We then retrieve the neighborhood disadvantage value of the census tract that contains that centroid, and use that as the imputed disadvantage value for all the missing disadvantage values for that subject. Note that the activity space centroid may be in a census tract for which an observed activity space location occurs in the retained values, or it may be in an entirely new tract. Thus, unlike the HD imputation, the imputed neighborhood disadvantage value may be different than any of the observed disadvantage values contained in the data. Each of the two imputation procedures, HD and ASC, is applied to the 50% retained, 70% retained, and 90% retained data sets, resulting in six imputed data sets for analysis. Finally, using the simplest approach to deal with the missing data—listwise deletion—we use only the subsets of EMA observations that were non-missing in the 50%, 70%, and 90% retained data sets.

We report descriptive statistics for the neighborhood disadvantage variable for the original data set and for each of each of the six imputed data sets. We then report the Pearson correlation between the imputed disadvantage values and the observed disadvantage values from the original data set for the imputed observations (not including the retained observations) within each data set (50% imputed, 30% imputed, and 10% imputed). Because the data are clustered by subject, we also report the association between the imputed and original observed disadvantage values using GEE (clustered by subject) by regressing the original disadvantage value on the imputed value for the six data sets of imputed observations. The original disadvantage variable coefficient indicates the degree of association after controlling for subject-level clustering.

Using the imputed disadvantage values for each of the six imputed data sets, we recalculate the relative disadvantage variable values for the imputed EMA observations, and then re-fit Model 1. We also re-fit Model 1 to each of the three listwise-deleted data sets. We compare the resulting coefficients of the relative disadvantage variable derived using the six imputed and three listwise-deleted data sets to those from the original data set presented in Model 1 (Table 1). We then repeat the procedure applied to Model 1 to compare the coefficients of the moderating variable to those presented in Model 2 (Table 1). All procedures were carried out in ArcGIS (ESRI, Inc., Redlands, CA, USA) and SPSS (IBM, Inc., Armonk, NY, USA).

## 3. Results

### 3.1. Descriptive Statistics

The original data set contains 1617 EMA observations for 137 subjects, with a mean of 12 EMA observations per subject. However, the number of EMA observations varies widely among subjects, from 1 to 54. Table 2 shows the descriptive statistics for the original observed neighborhood disadvantage variable and the imputed disadvantage variable in each of the six imputed data sets. Note that the minimum and maximum values are identical for all data sets, but the mean and standard deviation vary. The number of valid *N* (number of EMA observations) and number of subjects also varies. This is because for a handful of subjects with low numbers of EMA observations available, there are not any EMA observations in the retained portion of the data set that can be used in the HD or ASC imputation procedures. This is the case for 10 subjects with 16 EMA observations in the 50% retained data set, and for five subjects with eight EMA observations in the 70% retained data subset. In addition, the ASC imputation resulted in one imputed activity space location in a census tract with a zero population; thus, the disadvantage value for this EMA observation could not be imputed. Not surprisingly, the mean values for the 50% retained data set depart the most from the original observed disadvantage mean due to the larger percentage of missingness. The HD imputations maintain standard deviation values more similar to that of the original data as compared to the ASC imputations, particularly for data sets with a greater proportion of missing data.

### 3.2. Correlation of Observed and Imputed Neighborhood Disadvantage for Different Imputation Methods

**Figure 2:**
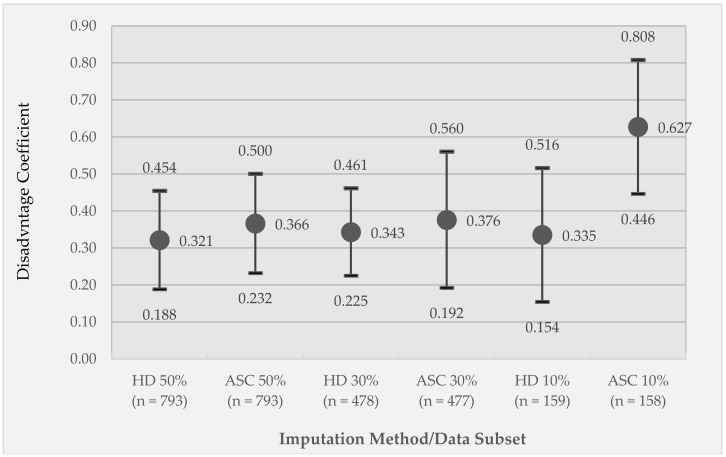
Generalized estimating equations (GEE) coefficients of observed disadvantage (clustered by subject) regressed on imputed disadvantage values. Coefficients are reported for each combination of imputation technique (hot deck (HD) or activity space centroid (ASC)) applied to different subsets of the imputed data (50% imputed, 30% imputed, and 10% imputed). Circle markers represent coefficient values; whiskers represent 95% confidence intervals. All coefficients are significant at *p* < 0.005.

Here, we report Pearson *r* correlations of the original, observed disadvantage with the imputed disadvantage values for subsets of the data that were imputed (not including the retained data). For the 50% imputed data subset, *r* = 0.25 and *r* = 0.34 for the HD (*n* = 793) and ASC (*n* = 793) imputation methods, respectively. For the 30% imputed data subset, *r* = 0.38 (*n* = 478) and *r* = 0.33 (*n* = 477), for HD and ASC respectively. For the 10% imputed data subset, *r* = 0.32 (*n* = 159) and *r* = 0.53 (*n* = 158), for HD and ASC respectively. All correlations are significant at *p* < 0.005. Results of the GEE of observed disadvantage regressed on imputed disadvantage, controlling for clustering by subject, are reported in Figure 2. When adjusted by subject-level clustering, ASC tends to outperform HD imputation across all three imputed data sets, though the difference is modest for the 50% and 30% imputed data sets.

### 3.3. Comparison of Model 1 GEE Relative Disadvantage Coefficients between the Original Data Set and the Listwise Deletion and Imputed Data Sets

**Figure 3:**
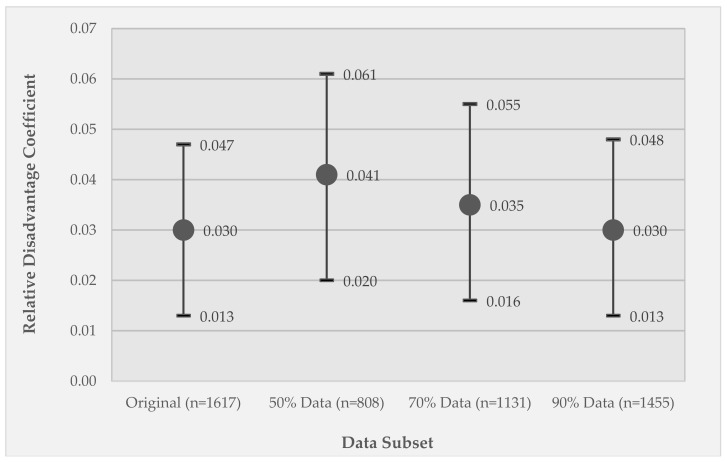
Results for the listwise deletion data sets for Model 1, GEE coefficients of the effect of relative disadvantage on momentary stress. Coefficients are reported for the original data set (100% retained) and each data subset—50% retained, 70% retained, and 90% retained subsets of the original data set. Circle markers represent coefficient values; whiskers represent 95% confidence intervals. All coefficients are significant at *p* < 0.005.

Figure 3 shows the results of Model 1 applied to the original data compared to the listwise deletion approach, in which the model is applied to 50%, 70%, and 90% of the EMA observations retained from the original data set. The relative disadvantage coefficient value is shown, along with 95% confidence intervals. Not surprisingly, a greater percentage of missing data results in larger departures of the coefficient value from that derived from the original data set. When 50% of the values are missing, the resulting estimate departs from the original coefficient estimate by approximately one third.

**Figure 4:**
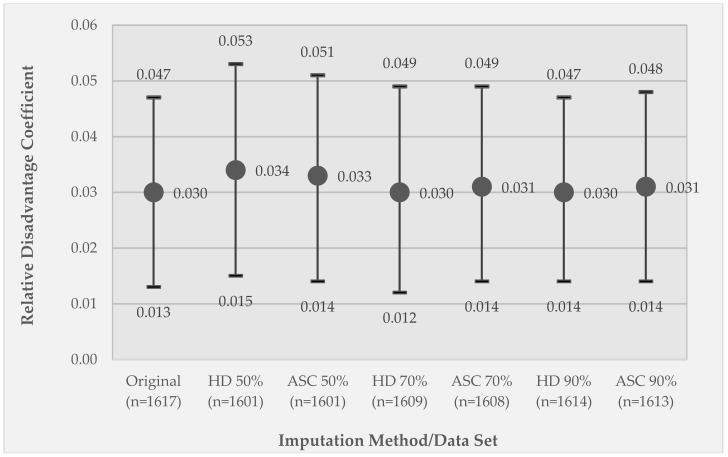
Results for the imputed data sets for Model 1, GEE coefficients of the effect of relative disadvantage on momentary stress. Coefficients are reported for the original data set (100% retained) and for each imputation technique (hot deck (HD) or activity space centroid (ASC)) applied to different percentages of the retained observations (50% retained (50% imputed), 70% retained (30% imputed), and 90% retained (10% imputed)). Circle markers represent coefficient values; whiskers represent 95% confidence intervals. All coefficients are significant at *p* < 0.005.

Figure 4 shows an analogous graph of the results of Model 1 comparing the original data set with different imputed data sets using the two imputation techniques and the different percentage of observations retained versus imputed. Departures from the original relative disadvantage coefficient value are modest, with the 50% retained data sets departing the most from the original coefficient value.

### 3.4. Comparison of Model 2 GEE Moderator Coefficients between the Original Data Set and the Listwise Deletion and Imputed Data Sets

**Figure 5:**
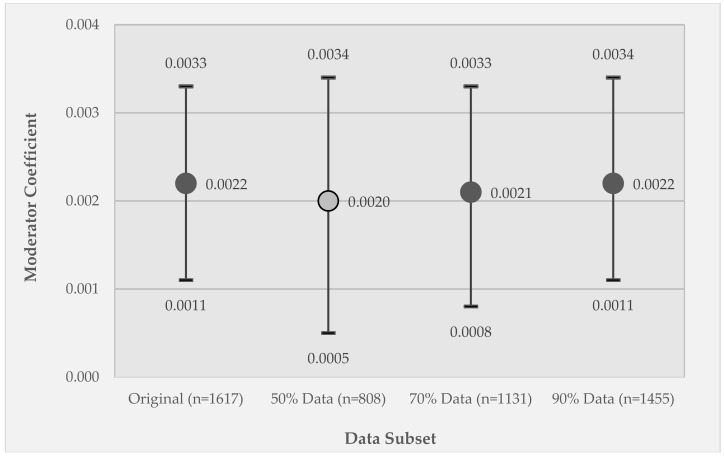
Results for the listwise deletion data sets for Model 2, GEE coefficients of the effect of the moderator variable (relative disadvantage X substance use) on momentary stress. Coefficients are reported for the original data set (100% retained) and for each data subset—50% retained, 70% retained, and 90% retained subsets of the original data set. Circle markers represent coefficient values; whiskers represent 95% confidence intervals. Marker fill indicates coefficient significance, where dark gray is *p* < 0.005 and light gray is *p* < 0.01.

**Figure 6:**
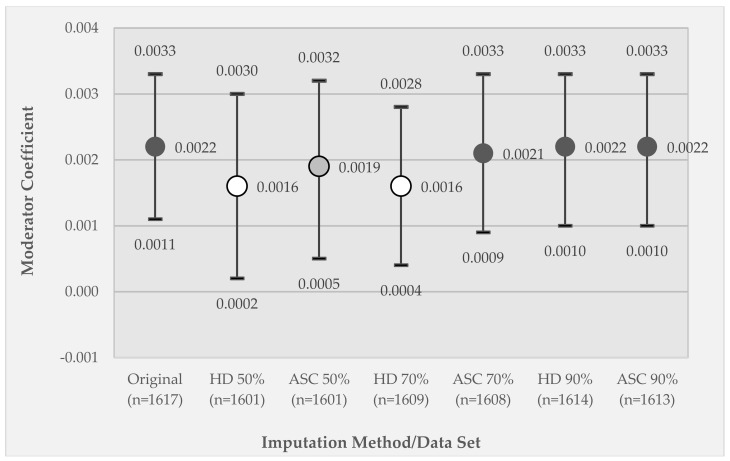
Results for the imputed data for Model 2, GEE coefficients of the effect of the moderator variable (relative disadvantage X substance use) on momentary stress. Coefficients are reported for the original data set (100% retained) and for each combination of imputation technique (hot deck (HD) or activity space centroid (ASC)) applied to different percentage retained data sets (50% retained (50% imputed), 70% retained (30% imputed), and 90% retained (10% imputed)). Circle markers represent coefficient values; whiskers represent 95% confidence intervals. Marker fill indicates coefficient significance, where dark gray is *p* < 0.005, light gray is *p* < 0.01, and white is *p* < 0.05.

Figure 5 and Figure 6 present results analogous to those presented in Figure 3 and Figure 4, but for the coefficient estimate for the moderator variable (substance use X relative disadvantage) in Model 2. In the results for the listwise deletion comparisons (Figure 5), the coefficient values are relatively stable across the different data subsets, but the coefficient for the 50% data subset changes in significance from *p* < 0.005 to *p* < 0.01 and, notably, the confidence intervals are substantially larger due to the substantial reduction in sample size.

In Figure 6, which compares results across the six different imputed data sets, substantial differences are observed. The coefficient significance is reduced to *p* < 0.05 for the 50% retained and 70% retained HD imputed data sets, and to *p* < 0.01 for the 50% retained ASC imputed data set due to the wider confidence intervals. For both the 50% and 70% retained data sets, the ASC imputed coefficient value is nearer to the original coefficient value as compared to the HD imputation. When only 10% of the data are missing, both imputation methods recover the estimate and its variability from the original data.

## 4. Discussion

To the best of our knowledge, this research represents the first study to develop and compare methods for geographic imputation of missing activity space data derived from GEMA. While we consider this a pilot study that implements relatively simple imputation techniques on a single empirical data set, this research provides a proof of concept for how geographic imputation methods can be adapted for GEMA activity space data and other activity space data generated from similar study designs. Our results suggest that geographic imputation can be used effectively to estimate the location of missing GEMA activity space data in studies of place effects on health.

In our analyses, we found that listwise deletion of missing activity space data, particularly at levels of 50% and 30% missing data, can bias estimates of place effects on health, in the present case, the effect of relative disadvantage on momentary stress. Listwise deletion changed the magnitude, significance, and/or standard errors of the relative disadvantage coefficient, particularly for 50% missingness and when estimating the significance and standard errors of the moderated effect. These impacts were ameliorated to some extent by using geographic imputation, particularly in mitigating the inflation of the standard errors, though results were more variable regarding the moderation effects. We found that ASC imputation generally outperformed HD imputation, though the difference was relatively modest.

We acknowledge several limitations. Our analysis focused on a single empirical GEMA data set gathered from a study of neighborhood disadvantage, substance use, and psychological stress among a sample of urban adolescents. The generalizability of our findings to other data sets and analytical contexts is unknown. We also used only one random draw to distinguish between retained and deleted observations to test our imputation methods. A more robust approach would repeat the analysis multiple times using different randomly drawn retained and deleted observations. Further, we note that our description focuses on the imputation of the census tracts that contain the missing EMA locations, rather than the imputed activity space points themselves. However, this is for the purpose of testing the effects of imputation on the regression models; the HD and ASC imputation methods could just as easily be used to estimate the missing EMA locations at the point level by selecting the coordinate positions from the known EMA locations rather than the tract identifier.

We also acknowledge that there may be bias regarding which observations are missing. Many simple imputation procedures, including the ones developed in the present research, are themselves susceptible to bias when observations are not missing completely at random (MCAR) or missing at random (MAR) [48]. While this issue is germane to any study employing survey data, it is likely to be particularly problematic in GEMA, where individuals may be hesitant to reveal their location in studies of sensitive health behaviors, such as substance use or sexual behavior [50,51]. Indeed, individuals may purposively hide their location in certain situations. We should also note that the original EMA data contains extensive missing data; thus, the data set used to test the sensitivity of the listwise deletion and geographic imputation methods is itself potentially prone to these same issues of missing data.

It is also possible that missing location data may occur more often in certain environments, such as densely developed urban areas or indoors, where the GPS signal is more likely to be physically blocked. Notably, the limited research on this topic, including our own, has not found any substantial associations between the demographic characteristics of the subject or the environmental conditions of the location of data capture with the spatial accuracy or missingness of the EMA location data [21,42], though further research is certainly warranted. In future geographic imputation research, it may be useful to stratify the deleted observations by the environmental characteristics (e.g., by land use or degree of urbanization) of where the EMA location occurs, to investigate consequences of excluding EMA locations from certain types of environments.

The HD and ASC geographic imputation methods we developed for this pilot study are relatively simple and may be improved. First, the methods are ‘fixed’ or deterministic in the sense that they assign a single location to all missing activity space observations for each subject. This has the result of clustering all missing data at one location for each subject. One could introduce a stochastic element to geographic imputation [34,37,40], where, for instance, for each of a subject’s missing activity space locations, the HD imputation would randomly draw a new imputed location from the subject’s set of known locations, rather than ‘reuse’ a single imputed location. With this approach, locations visited more often would be more likely to be selected. Or, for the ASC imputation, instead of estimating all of a subject’s missing activity space locations to occur at the centroid of the known EMA locations, one could model the likelihood of the missing location occurring at a given location as a function of the distance to the centroid, generating a point cloud of estimated locations for missing activity space data dispersed around the centroid. This may address the issue of the centroid occurring in a tract outside the sample of known EMA locations. It may also be useful to exclude subjects with low numbers of known EMA locations to begin with, for instance, those subjects with less than five known EMA locations, thus ensuring an adequate sample of locations from which to base the imputation.

The geographic imputation methods described here could also be extended to model-based and multiple imputation approaches [30,31,32,33]. For example, a regression equation could be developed for each subject that estimates the likelihood that a particular tract (or other spatial encoding) contains a missing activity space point based on the distance of the tract to the subject’s home, the age and sex of the subject, and other characteristics of the individual. It may also be useful to incorporate the temporal component of the EMA data, such as the time of day or day of the week that the EMA response occurred, as the daily and weekly rhythms of life make it more likely for individuals to travel to certain locations on, say, weekdays versus weekends or mornings versus evenings. Such a model could also be improved by incorporating spatial and temporal dasymetric methods [52], where spatial partitions, such as land use or neighborhood socioeconomic characteristics, and temporal partitions, such as weekday/weekend divisions, can be used to further refine the model, as has been used with spatial data in previous geographic imputations of residential addresses [34,41,42]. This approach would yield a surface of the spatial distribution of the likelihood of a particular missing activity space location occurring, which could be used to stochastically generate a set of imputed values for each missing activity space location, which could in turn be used to generate a distribution of model coefficients that could then be pooled, as in conventional multiple imputation.

## 5. Conclusions

Health researchers utilizing GEMA or related approaches to collecting activity space data should be aware of the issue of missing location data and its impact on statistical analyses of place effects on health behaviors and outcomes. Geographic imputation can be used to estimate missing activity space locations and thus maintain statistical power and reduce bias in estimates of coefficients and standard errors in models of direct and moderated effects. The present pilot study provides an empirical example of both the impact of listwise deletion on analytical results and the implementation of two simple geographic imputation techniques adapted for GEMA activity space data. These geographic imputation techniques may be extended in future research by incorporating approaches from the non-geographic imputation literature as well as from conventional geographic imputation and spatial interpolation research that focus on non-activity space data.

## Figures and Tables

**Table 1:** Generalized estimating equations (GEE) models of momentary stress (*n* = 1617). Model 1 shows the direct effect of relative disadvantage and Model 2 shows the moderation of that effect by substance use.

Independent Variable	Model 1	Model 2
Intercept	1.872 *** (1.648–2.096)	1.753 *** (1.534–1.973)
Age 14 (Ref = Age 13)	0.094 (−0.197–0.384)	0.090 (−0.196–0.376)
Male (Ref = Female)	−0.168 (−0.449–0.113)	−0.184 (−0.460–0.091)
White (Ref = Af. American)	−0.569 *** (−0.959–(−0.188))	−0.422 * (−0.789–(−0.056))
Other Race (Ref = Af. American)	0.169 (−0.286–0.624)	0.316 (−0.146–0.778)
Substance Use	0.027 *** (0.017–0.037)	0.001 (−0.020–0.017)
Relative Disadvantage	0.030 *** (0.013–0.047)	0.031 *** (0.021–0.041)
Sub. Use X Rel. Disadvantage		0.002 *** (0.001–0.003)

*** *p* < 0.005, * *p* < 0.05.

**Table 2:** Descriptive statistics for observed and imputed neighborhood disadvantage using different imputation methods and for different data sets (HD = hot deck imputation; ASC = activity space centroid imputation; and 50%, 70%, and 90% refer to the percentage of the data retained versus imputed).

Variable	Valid *N* (# Subjects)	Minimum	Maximum	Mean (SD)
Original Disadvantage	1617 (137)	−18.54	13.47	−1.68 (7.30)
HD 50% Disadvantage	1601 (127)	−18.54	13.47	−2.77 (7.10)
ASC 50% Disadvantage	1601 (127)	−18.54	13.47	−1.97 (6.93)
HD 70% Disadvantage	1609 (132)	−18.54	13.47	−1.60 (7.33)
ASC 70% Disadvantage	1608 (132)	−18.54	13.47	−1.85 (6.96)
HD 90% Disadvantage	1614 (135)	−18.54	13.47	−1.89 (7.36)
ASC 90% Disadvantage	1613 (135)	−18.54	13.47	−1.62 (7.21)
